# Machine learning reveals sex-specific associations between cardiovascular risk factors and incident atherosclerotic cardiovascular disease

**DOI:** 10.1038/s41598-023-36450-4

**Published:** 2023-06-08

**Authors:** Soongu Kwak, Hyun-Jung Lee, Seungyeon Kim, Jun-Bean Park, Seung-Pyo Lee, Hyung-Kwan Kim, Yong-Jin Kim

**Affiliations:** 1 Division of Cardiology, Department of Internal Medicine, Seoul National University Hospital, Seoul National University College of Medicine, Daehak-ro 101, Jongno-gu, Seoul, 03080 Republic of Korea; 2 Division of Cardiology, Severance Cardiovascular Hospital, Yonsei University College of Medicine, 50-1 Yonsei-ro, Seodaemun-gu, Seoul, 03722 Republic of Korea; 3 College of Pharmacy, Dankook University, Dandae-ro 119, Dongnam-gu, Cheonan-si, Chungcheongnam-do 31116 Republic of Korea

**Keywords:** Cardiology, Risk factors

## Abstract

We aimed to investigate sex-specific associations between cardiovascular risk factors and atherosclerotic cardiovascular disease (ASCVD) risk using machine learning. We studied 258,279 individuals (132,505 [51.3%] men and 125,774 [48.7%] women) without documented ASCVD who underwent national health screening. A random forest model was developed using 16 variables to predict the 10-year ASCVD in each sex. The association between cardiovascular risk factors and 10-year ASCVD probabilities was examined using partial dependency plots. During the 10-year follow-up, 12,319 (4.8%) individuals developed ASCVD, with a higher incidence in men than in women (5.3% vs. 4.2%, *P* < 0.001). The performance of the random forest model was similar to that of the pooled cohort equations (area under the receiver operating characteristic curve, men: 0.733 vs. 0.727; women: 0.769 vs. 0.762). Age and body mass index were the two most important predictors in the random forest model for both sexes. In partial dependency plots, advanced age and increased waist circumference were more strongly associated with higher probabilities of ASCVD in women. In contrast, ASCVD probabilities increased more steeply with higher total cholesterol and low-density lipoprotein (LDL) cholesterol levels in men. These sex-specific associations were verified in the conventional Cox analyses. In conclusion, there were significant sex differences in the association between cardiovascular risk factors and ASCVD events. While higher total cholesterol or LDL cholesterol levels were more strongly associated with the risk of ASCVD in men, older age and increased waist circumference were more strongly associated with the risk of ASCVD in women.

## Introduction

The global burden of atherosclerotic cardiovascular disease (ASCVD) is increasing^1^. Primary prevention, which includes the control of cardiovascular risk factors through lifestyle modifications or pharmacotherapy to prevent the first occurrence of ASCVD, is essential to minimize cardiovascular mortality and morbidity. Importantly, the target of these interventions is based on the individualized probabilities of ASCVD events^2,3^. Therefore, accurate risk prediction is important for identifying high-risk individuals to maximize the benefits of primary prevention.

A body of evidence showed significant sex differences in the prevalence of cardiovascular risk factors and the incidence of ASCVD^4–11^. In the general population, men tend to have a higher prevalence of obesity, smoking, high blood pressure (BP), diabetes mellitus, and dyslipidemia when compared with women^4,5^. Regarding cardiovascular outcomes, men are at a higher risk of ischemic heart disease and cardiovascular mortality than women^6–11^. The associations between cardiovascular risk factors and outcomes also differ by sex^6–9^. These emphasize the importance of sex-specific cardiovascular risk assessment and targeted primary prevention strategies.

Several studies have shown that machine learning models have a similar or higher performance for predicting ASCVD probabilities compared to established risk scoring systems, such as the pooled cohort equations (PCE) or Framingham Risk Score^12–16^. However, few studies have developed sex-specific machine learning models. In addition, previous studies have rarely provided information on how each variable is associated with the outcome in these models, information which could significantly improve model interpretability. Thus, constructing separate machine learning models by sex and delineating the impact of cardiovascular risk factors on outcomes in these models may provide a deeper insight into the importance of cardiovascular risk assessment by sex.

We aimed to investigate the sex differences in cardiovascular risk factors and their association with outcomes using nationwide health examination data with a machine learning approach. The aims of this study were: (1) to develop a sex-specific machine learning model for the prediction of ASCVD probabilities; (2) to stratify important predictors of ASCVD in each sex in the machine learning models; (3) to investigate the sex-specific associations of these risk factors with ASCVD.

## Methods

### Cohort characteristics

The National Health Insurance Service (NHIS) in Korea covers the entire Korean population, and the NHIS database incorporates detailed information on the individuals’ sociodemographics, medical check-up results including laboratory tests and health behaviors, healthcare utilization including diagnoses and treatments, and date and causes of death^17^. The representative sample of this database has been made publicly available for researchers, and its validity as a reliable data source has been established^18^.

Specifically, this study utilized the ‘medical check-up sample cohort’ of the NHIS database, which includes approximately 510,000 randomly sampled individuals (10%) aged 40 years or older from the general Korean population who underwent the standardized national medical check-up program in 2002 or 2003. These individuals were recommended to undergo repeated biannual medical check-ups up to 2013. Of these, we selected individuals who underwent medical check-up in 2009 or 2010, as 2009 was when levels of not only total cholesterol but its individual components, including low-density lipoprotein (LDL) cholesterol, high-density lipoprotein (HDL) cholesterol, and triglycerides, were also measured. The date of the NHIS medical check-up in 2009/2010 was used as the index date for each individual. We excluded individuals with a previous history of cardiovascular diseases at the index date, including ischemic heart disease (International Classification of Diseases, Tenth Revision [ICD-10] codes I20–I25), heart failure (ICD-10 codes I50 and I420), stroke (ICD-10 codes I60–69), and atrial fibrillation (ICD-10 code I48). Other exclusion criteria were chronic obstructive pulmonary disease, liver cirrhosis, end-stage renal disease, and cancer.

This study conformed to the Declaration of Helsinki and the Institutional Review Board approved the study protocol (Seoul National University Hospital, approval number: E-2104-087-1211). The need for informed consent was waived by the same ethics committee (Institutional Review Board of Seoul National University Hospital) as anonymized data were used.

### Variable definitions

All clinical information was collected from the medical check-up conducted on the index date. Systolic and diastolic BP were measured after resting for at least 5 min. Data on smoking status, alcohol consumption, physical activity, and income levels were collected from structured self-administered questionnaires. Low income was defined as that within the lowest 30% of entire Korean residents. The lifetime amount of smoking was calculated as pack-years, and the mean alcohol consumption per day (g/day) was reported. The workload of daily physical activity was calculated as the metabolic equivalent of tasks (MET) minutes per week^19^, and the intensity of physical activity was categorized as low, moderate, and high intensity according to the International Physical Activity Questionnaire scoring protocol.

Past medical history of hypertension was defined as either (1) previous diagnostic codes for hypertension (ICD-10 codes I10–I13, I15) with prescription records of anti-hypertensive medications including angiotensin-converting enzyme inhibitors, angiotensin II receptor blockers, calcium channel blockers, thiazides, and beta-blockers, or (2) systolic/diastolic BP ≥ 140/90 mmHg measured at the medical check-up. A history of diabetes mellitus was defined by one of the followings: (1) previous diagnostic codes for diabetes mellitus (ICD-10 codes E11–E14) accompanied with prescription records of glucose-lowering medications, or (2) fasting glucose level > 126 mg/dL at the medical check-up. Dyslipidemia was defined as either (1) diagnostic codes for dyslipidemia (ICD-10 code E78) with prescription records of lipid-lowering medications or (2) total cholesterol ≥ 240 mg/dL at the medical check-up.

Laboratory results, including fasting glucose, cholesterol, and liver enzyme levels (aspartate transaminase [AST] and alanine aminotransferase [ALT]), were obtained from the tests conducted on the index date. The estimated glomerular filtration rate was calculated using the Chronic Kidney Disease Epidemiology Collaboration equation. Proteinuria was examined using the dipstick test and categorized as negative, trace, or positive. The PCE method was used to calculate the probabilities of 10-year ASCVD according to guidelines, using the original beta coefficients provided by the guideline^20^.

### Outcome assessment

Individuals were followed up from the index date to December 31st, 2019, or death, whichever came first. The median follow-up duration of study participants was 10.1 years (interquartile interval, 9.6–10.5 years).

The primary endpoint was newly developed 10-year ASCVD events, defined as a composite of myocardial infarction, stroke, heart failure, and cardiovascular death. Myocardial infarction was defined as hospital admission with a diagnosis of non-ST-elevation and ST-elevation myocardial infarction (ICD-10 codes I21 and I22). Stroke events were defined as a hospital admission with a diagnosis of ischemic or hemorrhagic stroke (ICD-10 codes I60–I64), along with brain computed tomography or magnetic resonance imaging during hospitalization. Heart failure was defined as hospitalization for heart failure (ICD-10 codes I50 and I42). Cardiovascular death was defined as mortality attributed to cardiovascular causes (ICD-10 codes for death: I00–I99).

### Random forest model

In this study, random forest models were developed to predict 10-year ASCVD probabilities. We chose the random forest model over other machine learning models because it can effectively handle high-dimensional non-linear data and has a reduced tendency to overfit, thereby generally yielding high prediction accuracy in large-scale clinical datasets^21,22^. Moreover, the random forest model provides an effective variable selection that estimates variable importance.

We included 16 variables in the random forest model development: age (years), body mass index (BMI) (kg/m^2^), waist circumference (cm), systolic BP (mmHg), diastolic BP (mmHg), smoking (pack-year), alcohol consumption (g/day), physical activity (MET minutes per week), fasting glucose (mg/dL), total cholesterol level (mg/dL), triglyceride (mg/dL), LDL cholesterol level (mg/dL), HDL cholesterol level (mg/dL), estimated glomerular filtration rate (mL/min/1.73m^2^), AST (IU/L), and ALT (IU/L). The inclusion criteria were established risk factors for adverse cardiovascular events and variables routinely assessed for cardiovascular risk prediction^2,3^. We included only continuous variables since the random forest model may be biased in the assessment of relative variable importance by the variable type^23^. We used the relevant continuous variables for the categorical type of cardiovascular risk factors (i.e., fasting glucose level instead of diabetes mellitus). In addition, including additional categorical variables in the model (i.e., proteinuria) did not significantly improve risk prediction.

The outcome for the random forest model was set as the 10-year ASCVD event. We constructed a separate random forest model for each sex, and each group of men and women was randomly divided into training (70%) and test (30%) sets, the commonly used division ratio in machine learning studies. A decision tree was grown, and a random set of variables was chosen to split the samples into two branches, maximizing the decrease in node impurity. The predicted probability was a numeric value that ranged from 0 to 1. The model performance was tested with a different number of decision trees (*ntree*), minimum value of terminal node size (*nodesize*), and the number of variables randomly sampled as candidates at each split (*mtry*) (Supplemental Table S1)^24^. The best-performing model with the highest area under the receiver operating characteristic curve (AUC) was selected as the final model. The performance of random forest models appeared robust with changes in the parameters: mean AUC values were between 0.721 (standard deviation 0.009) in men and 0.757 (standard deviation 0.013) in women. The calibration of random forest models was assessed to evaluate the relationship between predicted versus actual probabilities of 10-year ASCVD.

### Relative variable importance

We used the permutation variable importance to stratify the importance of predictors in the random forest model^25,26^. The difference in prediction error before and after randomly permuting each variable is calculated, which is averaged over all trees and normalized by the standard deviation. The resulting measure is reported as the mean decrease in accuracy. A greater mean decrease in accuracy indicates a higher level of variable importance in the respective random forest model.

### Partial dependency plot

For the top ten important variables, the relationship between the variables and 10-year ASCVD probabilities in the random forest model was visualized using the partial dependency plot, which is a useful tool to improve the model’s interpretability^27^. The partial dependency plot is generated by calculating the marginal effect of a variable of interest on the outcome and integrating out the effects of all other variables^28,29^. The average probabilities of 10-year ASCVD were calculated at different values of a variable, which were traced using locally estimated scatterplot smoothing curves. Partial dependency plots were compared between men and women to investigate whether there were significant sex differences in these associations. To verify the associations between variables and ASCVD probabilities on the partial dependency plots, we further performed conventional Cox analysis, confirming these associations and assessing for any sex differences.

### Statistical analysis

Continuous variables were presented as median values with interquartile ranges, and categorical variables were presented as frequencies with percentages. Differences between the groups were compared using the Kruskal–Wallis test for continuous variables and the chi-square test for categorical variables. The cumulative incidence of 10-year ASCVD and each component of the outcome were calculated using Kaplan–Meier estimates and compared between men and women using the log-rank test. The performance of the PCE-predicted ASCVD probabilities and random forest models was evaluated using AUC and compared using DeLong’s method.

The associations between the top ten important variables in the random forest model and the risk of 10-year ASCVD were examined using Cox proportional hazard analysis and were reported as hazard ratios (HRs) with 95% confidence intervals (CIs). In the Cox analysis, BMI was categorized into < 18.5, ≥ 18.5 to < 25, ≥ 25 to < 30, and ≥ 30 kg/m^2^ based on the U-shaped relationship between BMI and 10-year ASCVD probabilities observed in the partial dependency plot. Multivariable Cox models were adjusted for variables included in the PCE, avoiding multicollinearity. The Cox proportionality assumption was evaluated using the scaled Schoenfeld residuals plots. The differences in risks between men and women were tested in Cox models using the interaction term.

A two-tailed *P* < 0.05 was considered statistically significant. All analyses were performed using R version 3.3.0 (R Foundation for Statistical Computing, Vienna, Austria). The R package *randomForest* was used for model development, and the partial dependency plots were generated using the *pdp* package^29^.

## Results

### Baseline characteristics according to sex

Of the 258,279 individuals, 132,505 (51.3%) were men and 125,774 (48.7%) were women. Women were slightly older than men (56 vs. 55 years, *P* < 0.001) and had a lower BMI (Table 1). Men had a higher systolic and diastolic BP (both *P* < 0.001), with a higher prevalence of hypertension (35.5% vs. 33.6%, *P* < 0.001), but the use of antihypertensive medications was less frequent in men than in women (25.8% vs. 29.8%, *P* < 0.001). The proportion of current smokers and the amount of alcohol consumption were markedly higher in men than in women (current smokers: 31.2% vs. 1.5%, *P* < 0.001; alcohol consumption: 5.7 vs. 0.0 g per day, *P* < 0.001). Diabetes mellitus was also more prevalent in men than in women (12.6% vs. 8.6%, *P* < 0.001), with higher fasting glucose levels. On the other hand, women more frequently had dyslipidemia than men (26.6% vs. 17.9%, *P* < 0.001), and both total cholesterol and LDL cholesterol levels were significantly higher in women. The PCE-predicted 10-year ASCVD probabilities were 7.6% in men and 2.6% in women (*P* < 0.001).

**Table 1:** Baseline characteristics of the study participants by sex.

	Men (n = 132,505)	Women (n = 125,774)	*P*
Age, years	55 (50–62)	56 (52–64)	< 0.001
BMI, kg/m^2^	24.1 (22.3–25.8)	23.6 (21.8–25.6)	< 0.001
Waist circumference, cm	84 (80–89)	78 (73–84)	< 0.001
Systolic BP, mmHg	125 (117–135)	120 (110–132)	< 0.001
Diastolic BP, mmHg	80 (70–85)	77 (70–80)	< 0.001
Low income, n (%)	21,911 (16.6)	31,574 (25.1)	< 0.001
Residents in a capital metropolitan area n (%)	56,389 (42.6)	50,883 (40.5)	< 0.001
Current smoker	41,365 (31.2)	1,828 (1.5)	< 0.001
Smoking, pack-year	9.0 (0.0–21.0)	0.0 (0.0–0.0)	< 0.001
Alcohol consumption, gram per day	5.7 (0.0–20.0)	0.0 (0.0–0.0)	< 0.001
Physical activity score, METs times minutes per week	558 (160–973)	396 (0–758)	< 0.001
Intensity of physical activity			< 0.001
Low	64,553 (48.7)	69,851 (55.5)	
Moderate	54,893 (41.4)	46,986 (37.4)	
High	13,059 (9.9)	8937 (7.1)	
Past medical history, n (%)			
Hypertension	47,058 (35.5)	42,206 (33.6)	< 0.001
Diabetes mellitus	16,730 (12.6)	10,777 (8.6)	< 0.001
Dyslipidemia	23,709 (17.9)	33,486 (26.6)	< 0.001
Medications, n (%)			
Aspirin	13,857 (10.5)	13,311 (10.6)	0.302
Statin	9892 (7.5)	14,500 (11.5)	< 0.001
Antihypertensive	34,251 (25.8)	37,491 (29.8)	< 0.001
Laboratory exam			
Fasting glucose, mg/dL	97 (89–107)	93 (86–102)	< 0.001
Total cholesterol, mg/dL	196 (174–220)	205 (181–230)	< 0.001
Triglyceride mg/dL	125 (87–180)	107 (77–151)	< 0.001
LDL cholesterol, mg/dL	116 (95–138)	123 (102–147)	< 0.001
HDL cholesterol, mg/dL	50 (43–59)	55 (47–64)	< 0.001
Estimated glomerular filtration rate, mL/min/1.73m^2^	83.8 (72.8–95.1)	85.0 (72.9–97.2)	< 0.001
AST, IU/L	24 (20–30)	23 (19–27)	< 0.001
ALT, IU/L	23 (18–32)	18 (15–25)	< 0.001
Proteinuria			< 0.001
Negative	126,080 (95.2)	120,527 (95.8)	
Trace	3099 (2.3)	2504 (2.0)	
Positive	3326 (2.5)	2743 (2.2)	
10-year ASCVD probabilities by PCE, %	7.6 (4.4–13.2)	2.6 (1.3–6.3)	< 0.001

Values are given as n (%) or median (interquartile range).

*ASCVD* atherosclerotic cardiovascular disease, *ALT* alanine aminotransferase, *AST* aspartate transaminase, *BMI* body mass index, *BP* blood pressure, *HDL* high-density lipoprotein, *LDL* low-density lipoprotein, *PCE* pooled cohort equations.

### Cardiovascular outcomes according to sex

During the 10-year follow-up period, 12,319 patients (4.8%) developed ASCVD, and the annualized rate of ASCVD in the entire cohort was 4.96 cases per 1000 person-year. The events of 10-year ASCVD included 3413 myocardial infarctions (1.3%), 6951 heart failure events (2.7%), 1776 stroke events (0.7%), and 2115 cardiovascular deaths (0.8%) (Table 2). The cumulative incidence of ASCVD was significantly higher in men than in women (5.50 vs. 4.40 cases per 1000 person-year, *P* < 0.001). Men had a significantly higher incidence of myocardial infarction, heart failure, stroke, and cardiovascular death than women (all *P* < 0.050).

**Table 2:** The incidence of 10-year ASCVD by sex.

	Entire cohort (n = 258,279)		Men (n = 132,505)		Women (n = 125,774)		*P*^b^
	Events (%)	Incidence rate^a^	Events (%)	Incidence rate^a^	Events (%)	Incidence rate^a^	
10-year ASCVD	12,319 (4.8)	4.96	6988 (5.3)	5.50	5331 (4.2)	4.40	< 0.001
Myocardial infarction	3413 (1.3)	1.36	2275 (1.7)	1.78	1138 (0.9)	0.93	< 0.001
Heart failure	6951 (2.7)	2.78	3650 (2.8)	2.85	3301 (2.6)	2.71	0.036
Cerebrovascular event	1776 (0.7)	0.71	1001 (0.8)	0.78	775 (0.6)	0.63	< 0.001
Cardiovascular death	2115 (0.8)	0.84	1241 (0.9)	0.96	874 (0.7)	0.71	< 0.001

^a^Per 1000 person-year.

^b^Comparison between men and women by log-rank test.

*ASCVD* atherosclerotic cardiovascular disease.

### Performance of random forest model according to sex

**Figure 1:**
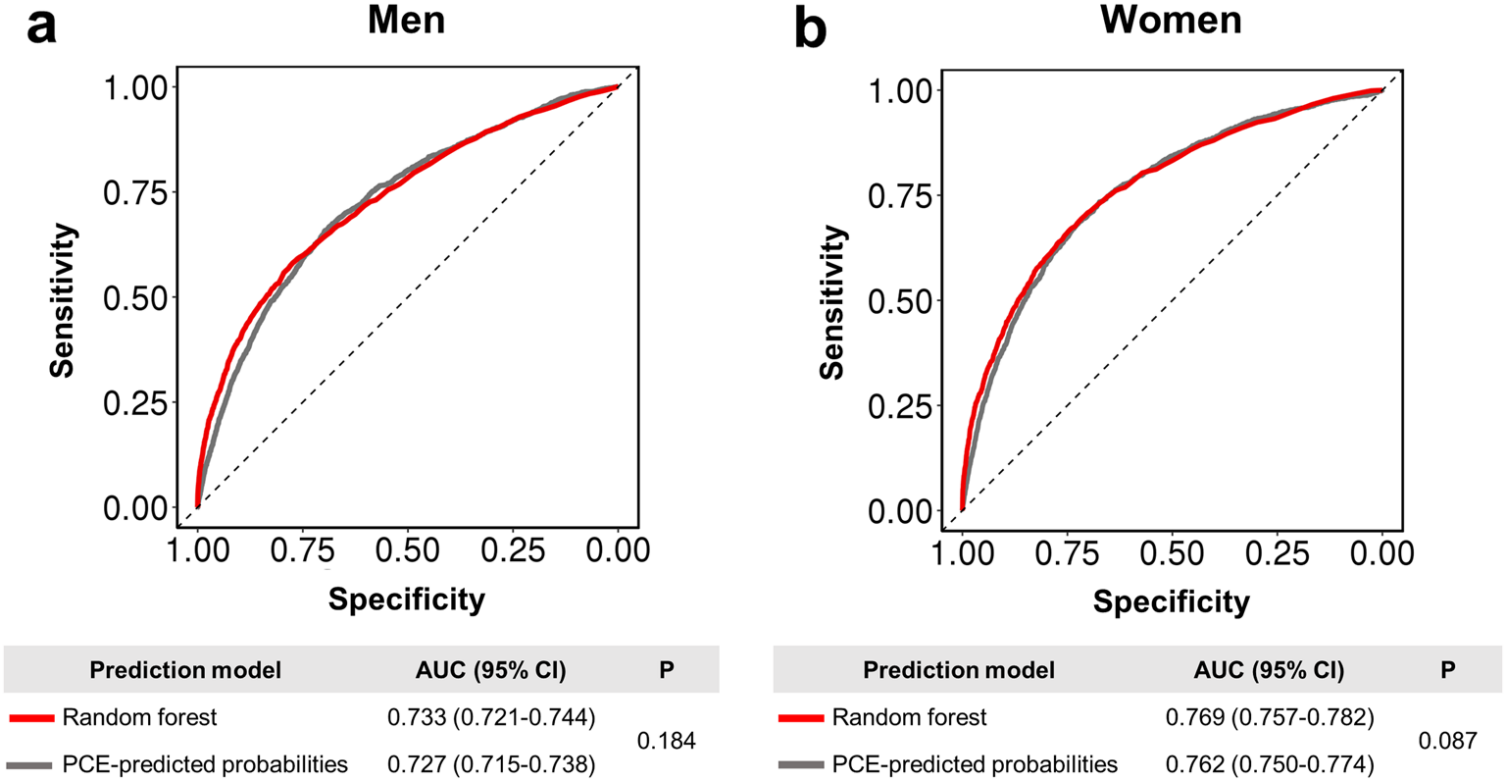
The area under the receiver operating characteristic curves of random forest models and PCE for predicting 10-year ASCVD according to sex. (**a**) Receiver operating characteristic curves in men. (**b**) Receiver operating characteristic curves in women. ^a^*P*-for-difference between the two curves. *ASCVD* atherosclerotic cardiovascular disease, *AUC* area under the receiver operating characteristic curves, *CI* confidence interval, *PCE* pooled cohort equations.

Figure 1 shows the performance of the random forest model and PCE-predicted ASCVD probabilities for the prediction of 10-year ASCVD. PCE showed fair predictability for 10-year ASCVD, with a higher AUC noted for women (men: AUC 0.727, 95% CI 0.715–0.738; women: AUC 0.762, 95% CI 0.750–0.774). Similarly, the random forest model achieved an AUC of 0.733 (95% CI 0.721–0.744) for men and a higher AUC of 0.769 (95% CI 0.757–0.782) for women. The performance between PCE-predicted ASCVD probabilities and the random forest model was similar in both sexes (P-for-difference = 0.184 in men and 0.087 in women). Calibration plots of the random forest models are presented in Supplemental Fig. S1. The ASCVD probabilities predicted by the random forest were generally similar to the observed probabilities, although there was a tendency towards underestimation of random forest models for high ASCVD probabilities in both men and women.

### Relative variable importance in random forest model according to sex

**Figure 2:**
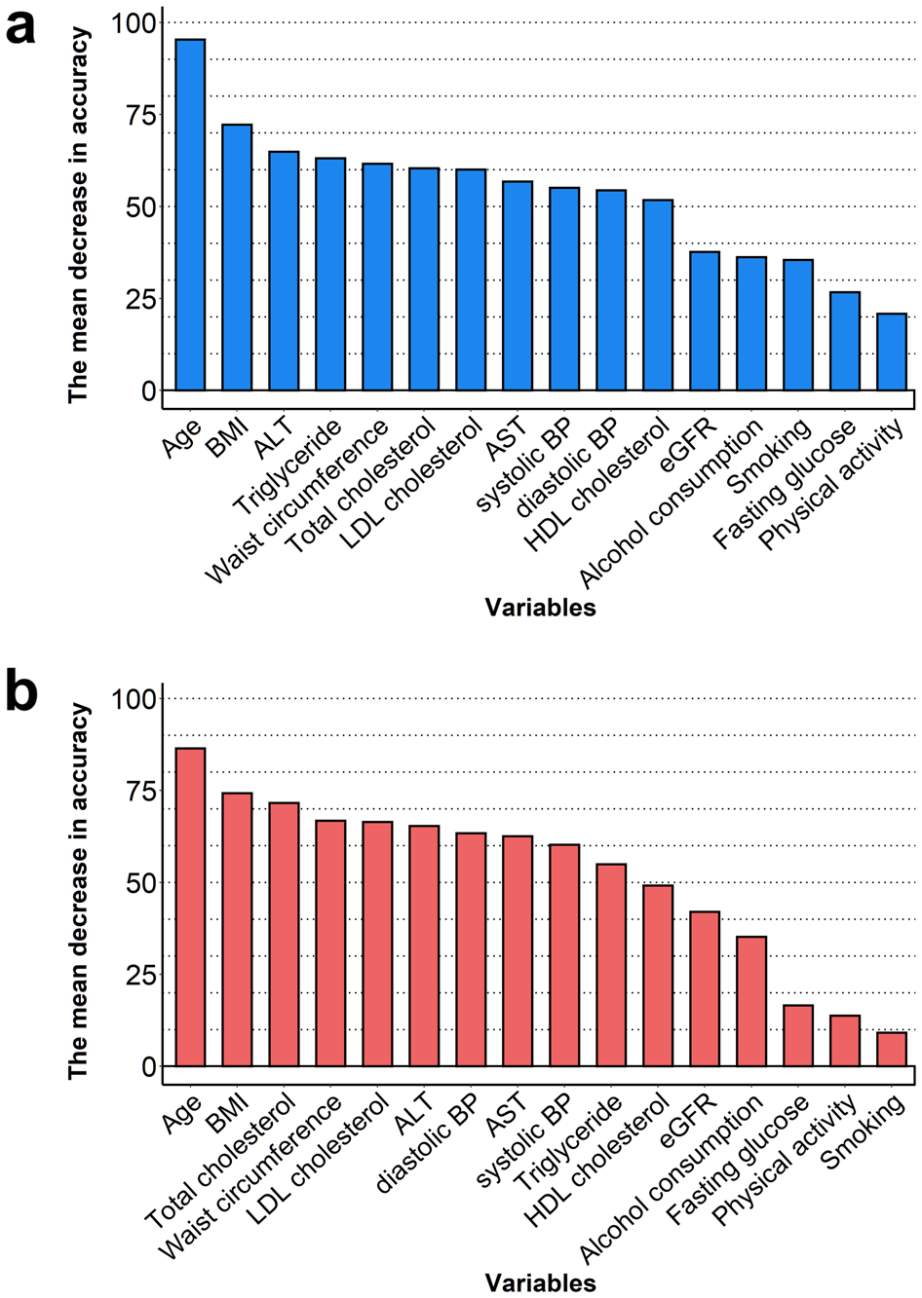
Relative variable importance of random forest model according to sex. The mean decrease in accuracy (permutation variable importance) was evaluated in random forest models of (**a**) men and (**b**) women. *ALT* alanine aminotransferase, *AST* aspartate transaminase, *BMI* body mass index, *BP* blood pressure, *eGFR* estimated glomerular filtration rate, *HDL* high-density lipoprotein, *LDL* low-density lipoprotein.

In both sexes, age and BMI were the two most important predictors in random forest models, with a mean decrease in accuracy of 95.4 and 72.2 in men and 86.5 and 74.3 in women (Fig. 2). Waist circumference, systolic BP, diastolic BP, total cholesterol, triglyceride, LDL cholesterol, AST, and ALT ranked between the third to tenth important variables in both sexes (Fig. 2). Smoking, drinking, physical activity, fasting glucose, HDL cholesterol, estimated glomerular filtration rate were variables with lower importance in both sexes. The top ten variables were consistently ranked between first and tenth place when different hyperparameters were used in the random forest model (Supplemental Fig. S2).

### Partial dependency plots of top ten important variables according to sex

**Figure 3:**
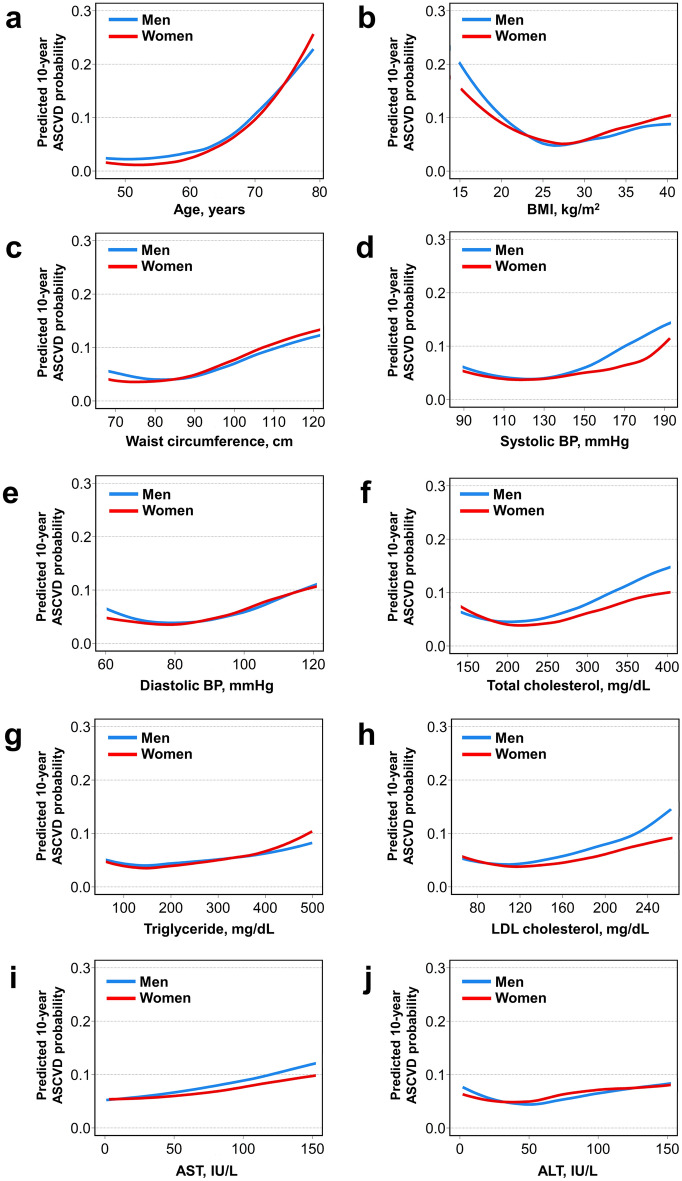
Partial dependency plots of top ten important cardiovascular risk factors in the random forest model according to sex. Partial dependency plots were generated from the final random forest models of men (blue) and women (red), which show the adjusted relationship between each variable and the predicted probabilities of ASCVD: (**a**) age, (**b**) BMI, (**c**) waist circumference, (**d**) systolic BP, (**e**) diastolic BP, (**f**) total cholesterol, (**g**) triglyceride, (**h**) LDL cholesterol, (**i**) AST, and (**j**) ALT. *ASCVD* atherosclerotic cardiovascular disease, *ALT* Alanine aminotransferase; AST, Aspartate transaminase; BMI, body mass index; BP, blood pressure; LDL, low-density lipoprotein.

Partial dependency plots, which show the adjusted relationship of variables with the outcome, were generated from the random forest models for men and women (Fig. 3). In the partial dependency plot, the probabilities of ASCVD increased with age, and this trend was more prominent in women than in men (Fig. 3a). There was a U-shaped relationship between BMI and ASCVD probabilities in both sexes (Fig. 3b). The ASCVD probabilities increased with a higher waist circumference, more strongly in women (Fig. 3c). For systolic BP, the ASCVD probabilities gradually increased beyond 140 mmHg in men, whereas it increased more steeply once the systolic BP exceeded approximately 170 mmHg in women (Fig. 3d). The probabilities of ASCVD gradually increased with higher diastolic BP in both sexes (Fig. 3e). Higher total cholesterol and LDL cholesterol levels were associated with increased probabilities of ASCVD more strongly in men, whereas the association between triglyceride and ASCVD appeared stronger in women (Fig. 3f–h). The increase in AST was more strongly associated with the ASCVD probabilities in men (Fig. 3i). ASCVD probabilities increased with higher ALT in both sexes (Fig. 3j).

### Associations of cardiovascular risk factors with 10-year ASCVD risk according to sex

In the univariable Cox analysis, an increase in age, BMI < 18.5 kg/m^2^ or ≥ 30 kg/m^2^ compared to BMI ≥ 18.5 to < 25 kg/m^2^, increase in waist circumference, systolic/diastolic BP, total cholesterol, triglyceride, AST was associated with higher ASCVD risk in both sexes (all *P* < 0.050) (Supplemental Table S2). However, an increase in LDL cholesterol or ALT level was associated with a higher ASCVD risk only in men.

In the multivariable Cox analysis, increased age was more strongly associated with a higher risk of 10-year ASCVD in women than in men (per 1 year increase, men: adjusted HR 1.09, 95% CI 1.09–1.10, *P* < 0.001; women: adjusted HR 1.11, 95% CI 1.10–1.11, *P* < 0.001, *P*-for-interaction by sex < 0.001) (Table 3). Higher waist circumference was associated with higher ASCVD risk more strongly in women (per 10 cm increase, men: adjusted HR 1.04, 95% CI 1.01–1.08, *P* = 0.015; women: adjusted HR 1.13, 95% CI 1.09–1.17, *P* < 0.001, *P*-for-interaction by sex < 0.001). In contrast, the increase in total cholesterol levels and LDL cholesterol levels were significantly associated with higher ASCVD risk only in men (total cholesterol, per 50 mg/dL increase, men: adjusted HR 1.17, 95% CI 1.13–1.21, *P* < 0.001, women: adjusted HR 1.03, 95% CI 0.99–1.07, *P* = 0.164, *P*-for-interaction by sex < 0.001) (LDL cholesterol, per 20 mg/dL increase, men: adjusted HR 1.06, 95% CI 1.04–1.07, *P* < 0.001, women: adjusted HR 1.00, 95% CI 0.99–1.02, *P* = 0.885, *P*-for-interaction by sex < 0.001).

**Table 3:** The associations of cardiovascular risk factors with the risk of 10-year ASCVD by sex.

	Men (n = 132,505)		Women (n = 125,774)		*P*-for-interaction by sex
	HR (95% CI)	*P*	HR (95% CI)	*P*	
Age^a^					
Per 1 year increase	1.09 (1.09–1.10)	< 0.001	1.11 (1.10–1.11)	< 0.001	< 0.001
BMI^b^					
≥ 18.5 to < 25 kg/m^2^	1.00 (ref)		1.00 (ref)		
< 18.5 kg/m^2^	1.64 (1.43–1.87)	< 0.001	1.37 (1.16–1.61)	< 0.001	0.336
≥ 25 to < 30 kg/m^2^	1.05 (1.00–1.11)	0.046	1.10 (1.04–1.17)	0.001	0.357
≥ 30 kg/m^2^	1.30 (1.11–1.52)	< 0.001	1.53 (1.36–1.72)	< 0.001	0.094
Waist^b^					
Per 10 cm increase	1.04 (1.01–1.08)	0.015	1.13 (1.09–1.17)	< 0.001	< 0.001
Systolic BP^a^					
Per 10 mmHg increase	1.08 (1.07–1.10)	< 0.001	1.08 (1.06–1.10)	< 0.001	0.181
Diastolic BP^c^					
Per 10 mmHg increase	1.09 (1.06–1.12)	< 0.001	1.11 (1.08–1.14)	< 0.001	0.125
Total cholesterol^d^					
Per 50 mg/dL increase	1.17 (1.13–1.21)	< 0.001	1.03 (0.99–1.07)	0.164	< 0.001
Triglyceride^b^					
Per 50 mg/dL increase	1.05 (1.03–1.06)	< 0.001	1.03 (1.01–1.05)	0.001	0.463
LDL cholesterol^a^					
Per 20 mg/dL increase	1.06 (1.04–1.07)	< 0.001	1.00 (0.99–1.02)	0.885	< 0.001
AST^b^					
Per 20 IU/L increase	1.11 (1.07–1.14)	< 0.001	1.05 (1.00–1.11)	0.047	0.199
ALT^b^					
Per 20 IU/L increase	1.05 (1.02–1.08)	0.003	1.02 (0.97–1.06)	0.483	0.125

All models were adjusted for variables included in the PCE, without multicollinearity.

^a^Age, systolic BP, and LDL cholesterol were adjusted for each other in addition to HDL cholesterol, diabetes mellitus, smoking status, use of anti-hypertensive medications, statin, and aspirin.

^b^Adjusted for age, systolic BP, LDL cholesterol, HDL cholesterol, diabetes mellitus, smoking status, use of anti-hypertensive medications, statin, and aspirin.

^c^Adjusted for age, LDL cholesterol, HDL cholesterol, diabetes mellitus, smoking status, use of anti-hypertensive medications, statin, and aspirin.

^d^Adjusted for age, systolic BP, HDL cholesterol, diabetes mellitus, smoking status, use of anti-hypertensive medications, statin, and aspirin.

*ASCVD* atherosclerotic cardiovascular disease, *ALT* alanine aminotransferase, *AST* aspartate transaminase, *BMI* body mass index, *CI* confidence interval, *HDL* high-density lipoprotein, *HR* hazard ratio, *LDL* low-density lipoprotein, *PCE* pooled cohort equations.

## Discussion

**Figure 4:**
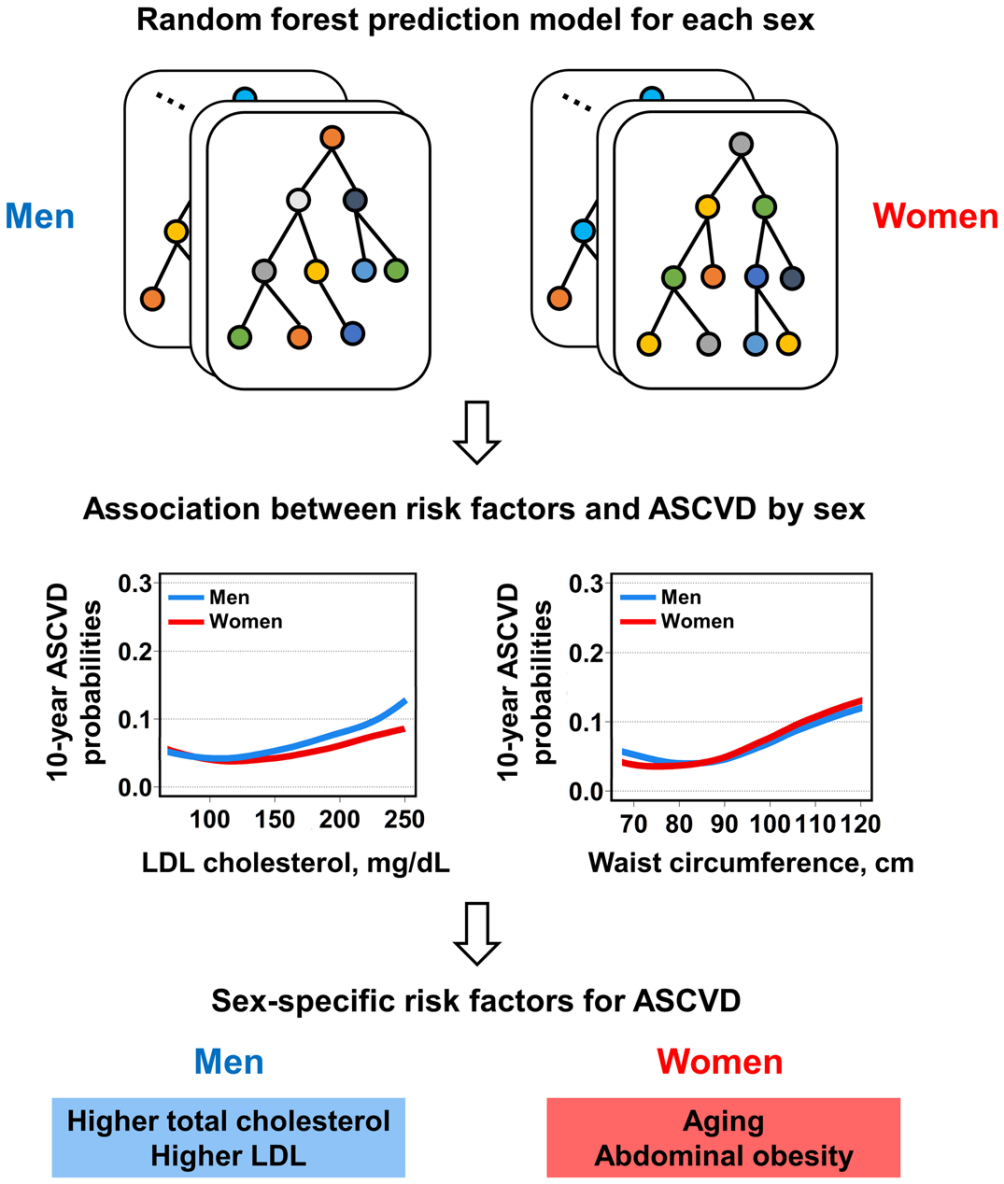
Graphical summary of study findings. *ASCVD* atherosclerotic cardiovascular disease, *LDL* low-density lipoprotein.

This study demonstrated significant sex differences in cardiovascular risk factors and their associations with ASCVD probabilities in the general population by applying machine learning to large-scale nationwide health examination data. Our random forest models had fair performance in predicting 10-year ASCVD, with a higher performance noted for women than for men. Importantly, the partial dependency plots demonstrated distinct sex-specific associations between these risk factors and 10-year ASCVD probabilities, which were also verified using Cox analysis. While the risk of ASCVD increased with higher total cholesterol and LDL cholesterol level more strongly in men, increased age and waist circumference were associated with higher ASCVD risk, especially in women (Fig. 4).

There are significant differences in the prevalence of cardiovascular risk factors according to sex. Men generally have more risk factors than women, including a higher prevalence of hypertension, smoking, and diabetes^4,5^, and this was also observed in our cohort. We also observed that the cumulative ASCVD events were significantly higher in men than in women, with more frequent events of myocardial infarction noted in men. Regarding pharmacologic treatment for ASCVD prevention, studies have shown that women may be less likely to be treated for dyslipidemia, whereas men may receive less antihypertensive treatment^4,5,30–32^. Patient perception of health status, patient-provider communications, and quality of life related to ASCVD may also be substantially different by sex^32^. Given these significant differences, sex-specific risk assessments and targeted prevention strategies are important to improve the outcomes of ASCVD.

While several previous studies have constructed machine learning model for predicting ASCVD, they rarely considered sex-specific models or evaluated sex-related differences using these models^12–16^. Interestingly, the performance of our random forest model was higher in women than in men (men: AUC 0.733 vs. women: AUC 0.769), and this was also observed for the PCE-predicted ASCVD probabilities (men: AUC 0.727 vs. women: AUC 0.762). This finding suggests there is a need to improve risk prediction specific to each sex. It may also imply more complex patterns and interactions between cardiovascular risk factors in men. Deep phenotyping or clustering individuals into similar but mutually exclusive subgroups may enable a more accurate prediction of ASCVD risk in men. The results also suggest that incorporating variables beyond the traditional cardiovascular risk factors may help to improve risk prediction for each sex. Genetic information, serum biomarkers, or socioeconomic factors, including income, education level, and relationship status, contribute to the development of ASCVD and have the potential to improve predictive ability^33,34^. For women, menopausal status or gestational diabetes are independent predictors of ASCVD^35^. Future studies are required to test these possibilities.

To enhance interpretability, we further investigated how each variable is associated with ASCVD probabilities using feature extraction technique of a partial dependency plot. In partial dependency plots of age, we observed that the adverse effects of aging on the risk of ASCVD were stronger in women than in men. The cardio-protective role of estrogen may be one reason for the heightened risk associated with aging in women, especially after menopause. Estrogen plays an important role in the maintenance of cardiac structure and function by reducing oxidative stress, preserving endothelial function, and preventing the accumulation of myocardial fibrosis^36^. Studies have shown that cardiovascular mortality rates increase more steeply with age in women than in men, especially after age 45–64 years^37^. Importantly, the earlier onset of natural menopause is associated with a higher risk of ASCVD, further supporting the concept of female vulnerability related to estrogen withdrawal^35,38^. However, our data did not contain information on the menopausal state or estrogen level, and future studies are warranted to clarify the role of estrogen and menopause in the sex-specific association between age and risk of ASCVD.

We observed a U-shaped association between BMI and ASCVD probability in both sexes. Recent studies have demonstrated that underweight is a robust risk factor for adverse cardiovascular events, including heart failure, cardiovascular mortality, and all-cause mortality^39,40^. The exact mechanism underlying the association between underweight and cardiovascular events is not yet fully understood. However, it is plausible to speculate that underweight may be indicative of malnutrition status or sarcopenia, both of which have significant implications for cardiovascular health. Our findings indicate that underweight is a significant risk factor in both men and women, highlighting the importance of optimizing body weight for the prevention of ASCVD.

Our study provides comprehensive information on the sex differences in cardiovascular risk factors and their association with the risk of ASCVD, which support the concept of targeted primary prevention interventions based on the assessment of individualized risk in terms of sex difference. Our findings imply that more active lipid-lowering therapy may benefit men, whereas control of abdominal obesity may be more crucial in women. However, more conclusive data demonstrating the benefits of such targeted intervention is eventually required, and future studies are warranted to test this hypothesis to improve cardiovascular outcomes in clinical practice.

### Limitations

Our study has several limitations. First, although our analysis showed associations between cardiovascular risk factors and outcomes, it does not demonstrate the cause-and-effect relationship. Potential confounders across biological and sociodemographic factors (i.e., income status) may have influenced the study results, which is inherent to all observational cohort studies. Second, some risk factors, such as BP, may change during follow-up, and the change or variability of risk factors was not considered in our analysis. Third, the random forest model is not designed to account for time-to-event survival data. While the random survival forest algorithm has been shown to provide risk prediction using this type of data, we were unable to implement this algorithm due to its lengthy computational time with large sample sizes. Lastly, our study was conducted solely on individuals of Korean ethnicity, which may limit the generalizability of our results to other populations. Notably, smoking and alcohol consumption were markedly lower in women compared to men, which is consistent with previous research in Korea^41,42^. Therefore, further research is necessary to determine if our findings are applicable to other ethnic groups.

## Conclusion

In conclusion, we developed a sex-specific machine learning model for predicting ASCVD events and investigated the associations between cardiovascular risk factors and ASCVD events in a large cohort from the general population using routine health examination data. While higher total cholesterol or LDL cholesterol levels were more strongly associated with the risk of ASCVD in men than in women, an increase in age and waist circumference were more strongly associated with the risk of ASCVD in women.

## Supplementary Information

Supplementary Information.

## Data Availability

All data created and/or used during this study are not publicly available according to the NHIS policy. Researchers can submit an application form through the NHIS website (https://nhiss.nhis.or.kr) to access and analyze the database.

## Abbreviations

ASCVD: Atherosclerotic cardiovascular disease
BMI: Body mass index
BP: Blood pressure
CI: Confidence interval
HDL: High-density lipoprotein
HR: Hazard ratio
ICD-10: International Classification of Diseases, Tenth Revision
LDL: Low-density lipoprotein
NHIS: National Health Insurance Service
PCE: Pooled cohort equations

## References

[1] Roth GA (2020). Global burden of cardiovascular diseases and risk factors, 1990–2019: Update from the GBD 2019 study. J. Am. Coll. Cardiol..

[2] Arnett DK (2019). 2019 ACC/AHA guideline on the primary prevention of cardiovascular disease: Executive summary: A report of the American College of Cardiology/American Heart Association Task Force on Clinical Practice Guidelines. J. Am. Coll. Cardiol..

[3] Lloyd-Jones DM (2019). Use of risk assessment tools to guide decision-making in the primary prevention of atherosclerotic cardiovascular disease: A special report from the American Heart Association and American College of Cardiology. J. Am. Coll. Cardiol..

[4] Peters SAE, Muntner P, Woodward M (2019). Sex differences in the prevalence of, and trends in, cardiovascular risk factors, treatment, and control in the United States, 2001 to 2016. Circulation.

[5] Pinho-Gomes AC, Peters SAE, Thomson B, Woodward M (2021). Sex differences in prevalence, treatment and control of cardiovascular risk factors in England. Heart.

[6] Millett ERC, Peters SAE, Woodward M (2018). Sex differences in risk factors for myocardial infarction: Cohort study of UK Biobank participants. BMJ.

[7] Albrektsen G (2016). Lifelong gender gap in risk of incident myocardial infarction: The Tromsø study. JAMA Intern. Med..

[8] Bots SH, Peters SAE, Woodward M (2017). Sex differences in coronary heart disease and stroke mortality: A global assessment of the effect of ageing between 1980 and 2010. BMJ Glob. Health.

[9] Jousilahti P, Vartiainen E, Tuomilehto J, Puska P (1999). Sex, age, cardiovascular risk factors, and coronary heart disease: A prospective follow-up study of 14 786 middle-aged men and women in Finland. Circulation.

[10] Mikkola TS, Gissler M, Merikukka M, Tuomikoski P, Ylikorkala O (2013). Sex differences in age-related cardiovascular mortality. PLoS ONE.

[11] Leening MJ, Ferket BS, Steyerberg EW, Kavousi M, Deckers JW, Nieboer D (2014). Sex differences in lifetime risk and first manifestation of cardiovascular disease: Prospective population based cohort study. BMJ.

[12] Cho SY (2021). Pre-existing and machine learning-based models for cardiovascular risk prediction. Sci. Rep..

[13] Weng SF, Reps J, Kai J, Garibaldi JM, Qureshi N (2017). Can machine-learning improve cardiovascular risk prediction using routine clinical data?. PLoS ONE.

[14] Rousset A (2021). Can machine learning bring cardiovascular risk assessment to the next level? A methodological study using FOURIER trial data. Eur. Heart J. Digit. Health.

[15] Steinfeldt J (2022). Neural network-based integration of polygenic and clinical information: Development and validation of a prediction model for 10-year risk of major adverse cardiac events in the UK Biobank cohort. Lancet Digit. Health.

[16] Nakanishi R (2021). Machine learning adds to clinical and CAC assessments in predicting 10-year CHD and CVD deaths. JACC Cardiovasc. Imaging.

[17] Choi E-K (2020). Cardiovascular research using the Korean national health information database. Korean Circ. J..

[18] Lee J, Lee JS, Park SH, Shin SA, Kim K (2017). Cohort profile: The national health insurance service-national sample cohort (NHIS-NSC), South Korea. Int. J. Epidemiol..

[19] Craig CL (2003). International physical activity questionnaire: 12-Country reliability and validity. Med. Sci. Sports Exerc..

[20] Goff DC (2014). 2013 ACC/AHA guideline on the assessment of cardiovascular risk: A report of the American College of Cardiology/American Heart Association Task Force on Practice Guidelines. Circulation.

[21] Touw WG (2013). Data mining in the life sciences with random forest: A walk in the park or lost in the jungle?. Brief Bioinform..

[22] Li J (2020). A multicenter random forest model for effective prognosis prediction in collaborative clinical research network. Artif. Intell. Med..

[23] Strobl C, Boulesteix AL, Zeileis A, Hothorn T (2007). Bias in random forest variable importance measures: Illustrations, sources and a solution. BMC Bioinform..

[24] Couronné R, Probst P, Boulesteix AL (2018). Random forest versus logistic regression: a large-scale benchmark experiment. BMC Bioinform..

[25] Breiman L (2001). Random forests. Mach. Learn..

[26] Austin AM (2022). Using a cohort study of diabetes and peripheral artery disease to compare logistic regression and machine learning via random forest modeling. BMC Med. Res. Methodol..

[27] Kwak S (2021). Markers of myocardial damage predict mortality in patients with aortic stenosis. J. Am. Coll. Cardiol..

[28] Jerome HF (2001). Greedy function approximation: A gradient boosting machine. Ann. Stat..

[29] Greenwell BM (2017). pdp: An R package for constructing partial dependence plots. R J..

[30] O'Meara JG, Kardia SL, Armon JJ, Brown CA, Boerwinkle E, Turner ST (2004). Ethnic and sex differences in the prevalence, treatment, and control of dyslipidemia among hypertensive adults in the GENOA study. Arch. Intern. Med..

[31] Choi HM, Kim HC, Kang DR (2017). Sex differences in hypertension prevalence and control: Analysis of the 2010–2014 Korea National Health and Nutrition Examination Survey. PLoS ONE.

[32] Okunrintemi V (2018). Gender differences in patient-reported outcomes among adults with atherosclerotic cardiovascular disease. J. Am. Heart Assoc..

[33] Colantonio LD (2017). Performance of the atherosclerotic cardiovascular disease pooled cohort risk equations by social deprivation status. J. Am. Heart Assoc..

[34] Yin X (2014). Protein biomarkers of new-onset cardiovascular disease: Prospective study from the systems approach to biomarker research in cardiovascular disease initiative. Arterioscler. Thromb. Vasc. Biol..

[35] El Khoudary SR (2020). Menopause transition and cardiovascular disease risk: Implications for timing of early prevention: A scientific statement from the American Heart Association. Circulation.

[36] Iorga A, Cunningham CM, Moazeni S, Ruffenach G, Umar S, Eghbali M (2017). The protective role of estrogen and estrogen receptors in cardiovascular disease and the controversial use of estrogen therapy. Biol. Sex Differ..

[37] Merz AA, Cheng S (2016). Sex differences in cardiovascular ageing. Heart.

[38] Muka T (2016). Association of age at onset of menopause and time since onset of menopause with cardiovascular outcomes, intermediate vascular traits, and all-cause mortality: A systematic review and meta-analysis. JAMA Cardiol..

[39] Held C (2022). Body mass index and association with cardiovascular outcomes in patients with stable coronary heart disease—A STABILITY substudy. J. Am. Heart Assoc..

[40] Lee HJ (2022). Age-dependent associations of body mass index with myocardial infarction, heart failure, and mortality in over 9 million Koreans. Eur. J. Prev. Cardiol..

[41] Kim I (2018). Comparison of district-level smoking prevalence and their income gaps from two national databases: The national health screening database and the community health survey in Korea, 2009–2014. J. Korean Med. Sci..

[42] Kim SY, Kim HJ (2021). Trends in alcohol consumption for Korean adults from 1998 to 2018: Korea national health and nutritional examination survey. Nutrients.

