# Supplementary material for: Machine learning reveals sex-specific associations between cardiovascular risk factors and incident atherosclerotic cardiovascular disease
Source: Sci Rep. 2023 Jun 8;13:9364. doi: 10.1038/s41598-023-36450-4 (PMC10250402; doi:10.1038/s41598-023-36450-4)
Supplement: Supplementary file 1 — Supplementary Information. [file 41598_2023_36450_MOESM1_ESM.docx]

**Supplemental Table S1. Hyperparameters tested in the development of a random forest model.**

|  | **Search grid** |
| --- | --- |
| Number of trees (*ntree*) | 250, 500, 1000 |
| Minimum value of terminal node size (*nodesize*) | 1, 5, 10, 50 |
| The number of variables randomly sampled as candidates (*mtry*) | 1, 2, 3, 4, 5 |

**Supplemental Table S2. Univariable Cox analysis of cardiovascular risk factors for the risk of 10-year ASCVD by sex.**

|  | **Men**  **(n=132,505)** | | **Women**  **(n=125,774)** | | **P-for-interaction by sex** |
| --- | --- | --- | --- | --- | --- |
|  | **HR (95% CI)** | **P** | **HR (95% CI)** | **P** |  |
| **Age** |  |  |  |  |  |
| per 1 year increase | 1.10 (1.09–1.10) | <0.001 | 1.12 (1.11–1.13) | <0.001 | <0.001 |
| **BMI** |  |  |  |  |  |
| ≥18.5 to <25 kg/m² | 1.00 (ref) |  | 1.00 (ref) |  |  |
| <18.5 kg/m² | 2.01 (1.76–2.30) | <0.001 | 1.62 (1.38–1.91) | <0.001 | 0.043 |
| ≥25 to <30 kg/m² | 1.02 (0.97–1.07) | 0.407 | 1.41 (1.33–1.49) | <0.001 | <0.001 |
| ≥30 kg/m² | 1.24 (1.06–1.44) | 0.006 | 2.15 (1.92–2.42) | <0.001 | <0.001 |
| **Waist** |  |  |  |  |  |
| per 10 cm increase | 1.15 (1.12–1.19) | <0.001 | 1.57 (1.52–1.62) | <0.001 | <0.001 |
| **Systolic BP** |  |  |  |  |  |
| per 10 mmHg increase | 1.17 (1.16–1.19) | <0.001 | 1.27 (1.25–1.29) | <0.001 | <0.001 |
| **Diastolic BP** |  |  |  |  |  |
| per 10 mmHg increase | 1.08 (1.05–1.10) | <0.001 | 1.28 (1.24–1.31) | <0.001 | <0.001 |
| **Total cholesterol** |  |  |  |  |  |
| per 50 mg/dL increase | 1.04 (1.01–1.08) | 0.020 | 1.04 (1.01–1.08) | 0.018 | 0.889 |
| **Triglyceride** |  |  |  |  |  |
| per 50 mg/dL increase | 1.04 (1.02–1.05) | <0.001 | 1.17 (1.15–1.19) | <0.001 | <0.001 |
| **LDL cholesterol** |  |  |  |  |  |
| per 20 mg/dL increase | 1.03 (1.02–1.04) | <0.001 | 1.00 (0.99–1.02) | 0.608 | 0.022 |
| **AST** |  |  |  |  |  |
| per 20 IU/L increase | 1.12 (1.08–1.15) | <0.001 | 1.16 (1.12–1.22) | <0.001 | 0.108 |
| **ALT** |  |  |  |  |  |
| per 20 IU/L increase | 0.97 (0.94–0.99) | 0.031 | 1.03 (0.99–1.07) | 0.215 | 0.022 |

ASCVD, atherosclerotic cardiovascular disease; ALT, Alanine aminotransferase; AST, Aspartate transaminase; BMI, body mass index; CI, confidence interval; HR, hazard ratio; LDL, low-density lipoprotein;

**Supplemental Figure S1. Calibration plots of random forest model in each sex.**

The predicted probabilities of 10-year ASCVD (x-axis) were plotted against the observed probability at 10-year (y-axis). The predicted probabilities were divided into 0.05 intervals, and the event rate of each bin was calculated. Crossbars indicate a 95% confidence interval. The dashed lines indicate the linear fit of the observations. The bar graphs at the bottom show the distribution of predicted probabilities in each group of men and women.


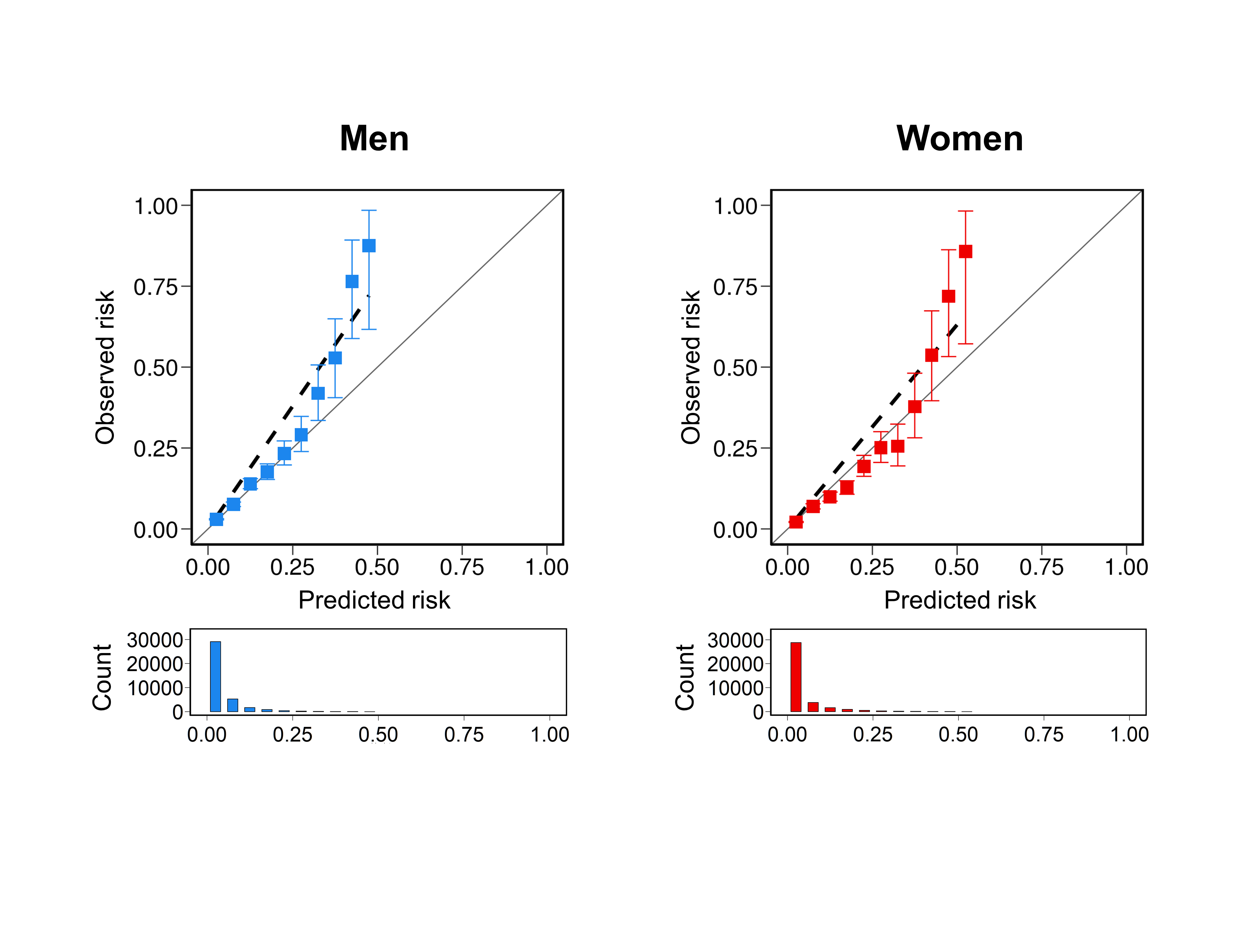


ASCVD, atherosclerotic cardiovascular disease

**Supplemental Figure S2. Assessment of variable importance in different hyperparameter settings.**

We tested whether the top ten important variables in the final random forest model were consistently identified when different hyperparameters were used. We conducted 60 iterations using different hyperparameters as shown in **Supplemental Table S1**.

**(a)** We calculated the percentage of each variable included in the top ten ranks across 60 iterations. Age, BMI, waist circumference, systolic BP, diastolic BP, total cholesterol, triglyceride, LDL cholesterol, AST, and ALT were almost always included in the top ten variables. However, smoking, drinking, physical activity, fasting glucose, HDL cholesterol, and estimated glomerular filtration rate were rarely included in the top ten variables.

*Variables are ordered in descending values of percentage for men.

**
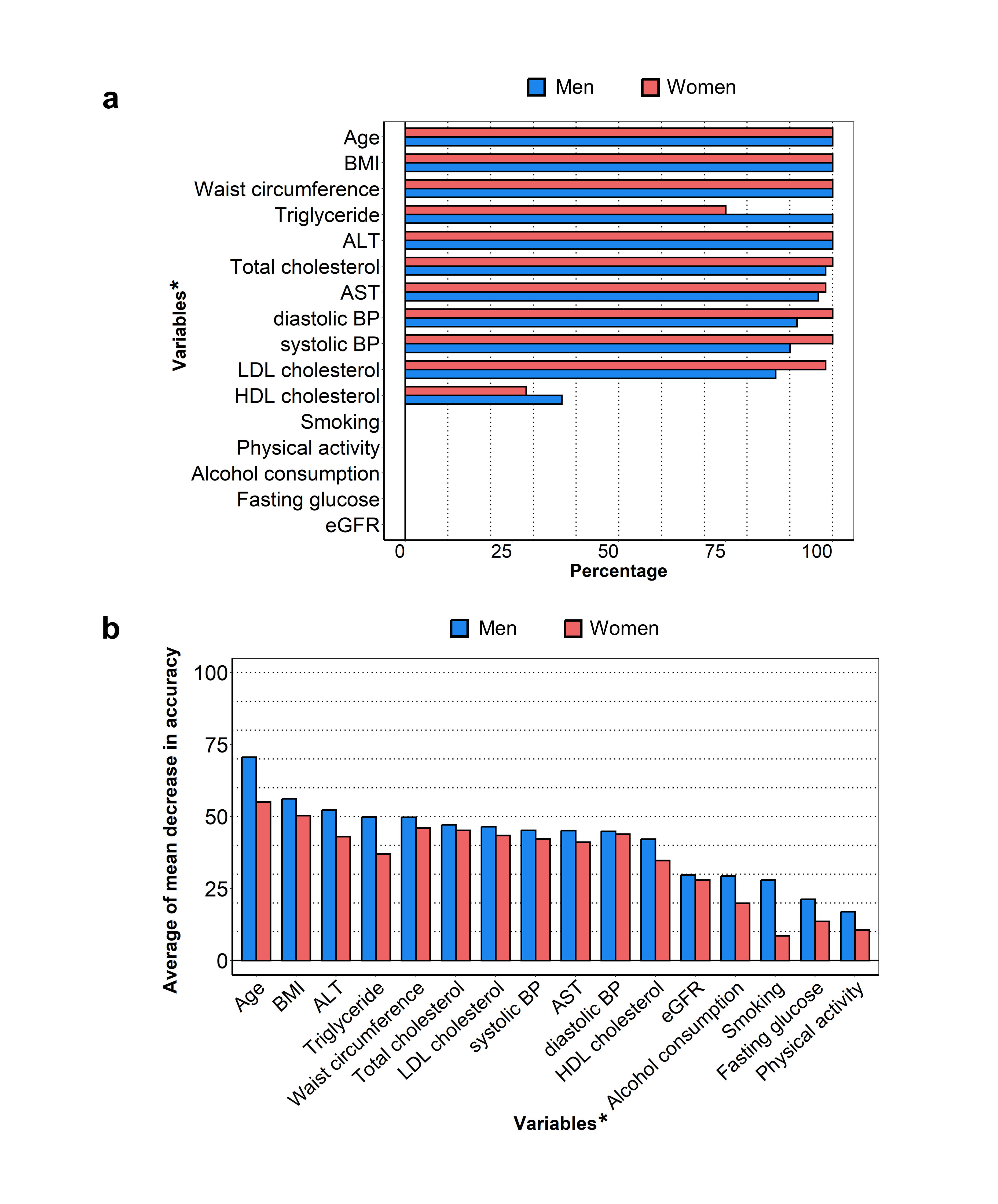
**

**(b)** The average mean decrease in accuracy across 60 iterations is shown. Again, smoking, drinking, physical activity, fasting glucose, HDL cholesterol, and estimated glomerular filtration rate showed a low average mean decrease in accuracy compared to the other 10 variables.

*Variables are ordered in descending values of averaged mean decrease in accuracy for men.

**
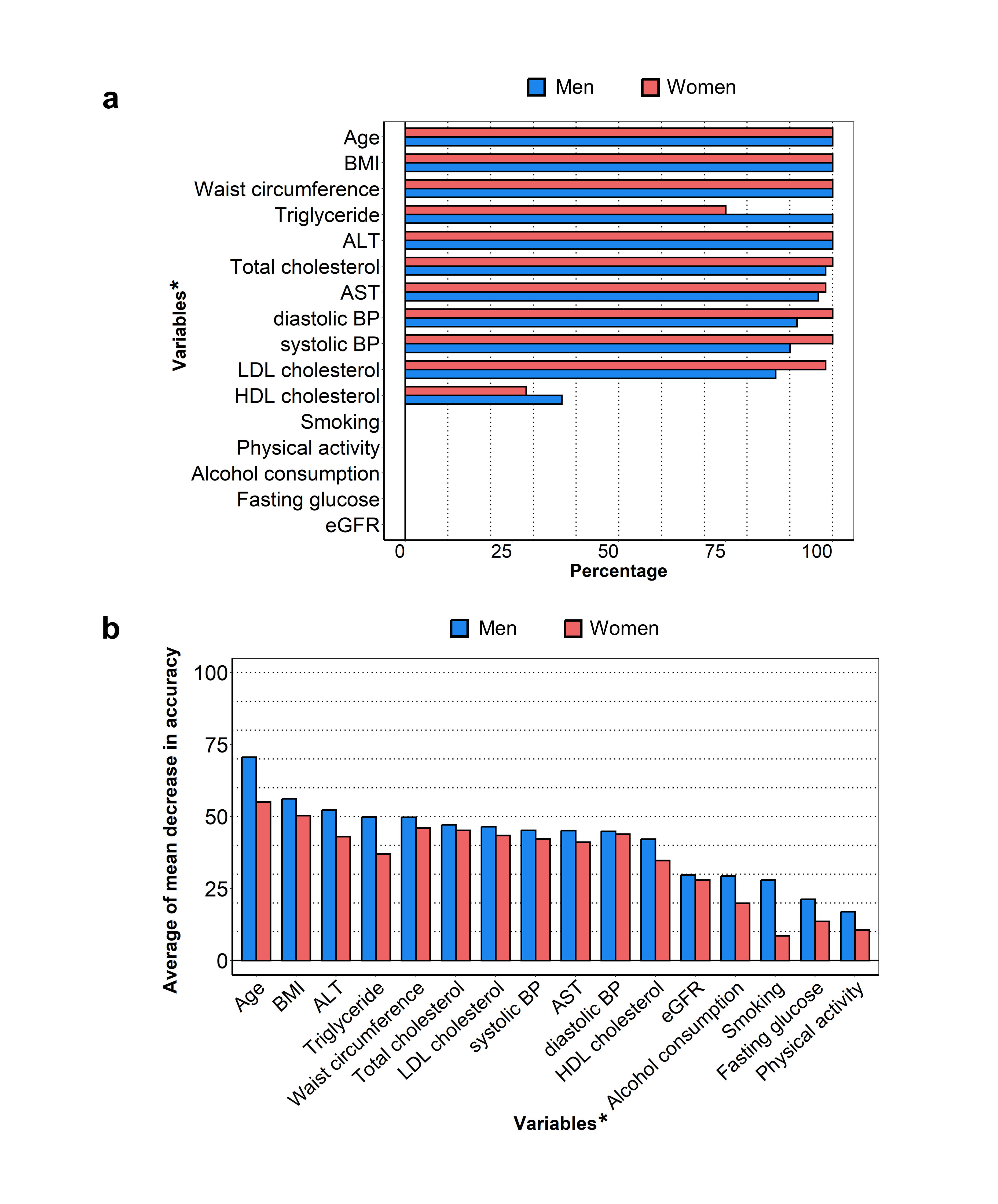
**

These findings indicate that the top ten important variables are consistently identified with different hyperparameter settings, demonstrating their robustness.

ALT, Alanine aminotransferase; AST, Aspartate transaminase; BMI, body mass index; BP, blood pressure; eGFR, estimated glomerular filtration rate; HDL, high-density lipoprotein; LDL, low-density lipoprotein
